# Butyrate prevents visceral adipose tissue inflammation and metabolic alterations in a Friedreich’s ataxia mouse model

**DOI:** 10.1016/j.isci.2023.107713

**Published:** 2023-08-28

**Authors:** Riccardo Turchi, Francesca Sciarretta, Veronica Ceci, Marta Tiberi, Matteo Audano, Silvia Pedretti, Concetta Panebianco, Valentina Nesci, Valerio Pazienza, Alberto Ferri, Simone Carotti, Valerio Chiurchiù, Nico Mitro, Daniele Lettieri-Barbato, Katia Aquilano

**Affiliations:** 1 Department Biology, University of Rome Tor Vergata, Rome, Italy; 2 IRCCS Fondazione Santa Lucia, Rome, Italy; 3 PhD Program in Evolutionary Biology and Ecology, Department of Biology, University of Rome Tor Vergata, Rome, Italy; 4 Laboratory of Resolution of Neuroinflammation, IRCCS Fondazione Santa Lucia, Rome, Italy; 5 DiSFeB, Dipartimento di Scienze Farmacologiche e Biomolecolari, Università degli Studi di Milano, Milan, Italy; 6 Gastroenterology Unit Fondazione IRCSS “Casa Sollievo della Sofferenza” Hospital San Giovanni Rotondo (FG)-Italy; 7 Department of Systems Medicine, University of Rome Tor Vergata, Rome, Italy; 8 Division of Experimental Neuroscience, IRCCS Fondazione Santa Lucia, Rome, Italy; 9 Institute of Traslational Pharmacology, IFT-CNR, Rome, Italy; 10 Microscopic and Ultrastructural Anatomy Research Unit, Department of Medicine and Surgery, Università Campus Bio-Medico di Roma, Rome, Italy; 11 Predictive Molecular Diagnostics, Fondazione Policlinico Universitario Campus Bio-Medico, Rome, Italy; 12 Department of Experimental Oncology, IEO, European Institute of Oncology IRCCS, Milan, Italy

**Keywords:** Pharmacology, Natural sciences, Biological sciences, Neuroscience

## Abstract

Friedreich’s ataxia (FA) is a neurodegenerative disease resulting from a mutation in the *FXN* gene, leading to mitochondrial frataxin deficiency. FA patients exhibit increased visceral adiposity, inflammation, and heightened diabetes risk, negatively affecting prognosis. We investigated visceral white adipose tissue (vWAT) in a murine model (KIKO) to understand its role in FA-related metabolic complications. RNA-seq analysis revealed altered expression of inflammation, angiogenesis, and fibrosis genes. Diabetes-like traits, including larger adipocytes, immune cell infiltration, and increased lactate production, were observed in vWAT. FXN downregulation in cultured adipocytes mirrored vWAT diabetes-like features, showing metabolic shifts toward glycolysis and lactate production. Metagenomic analysis indicated a reduction in fecal butyrate-producing bacteria, known to exert antidiabetic effects. A butyrate-enriched diet restrained vWAT abnormalities and mitigated diabetes features in KIKO mice. Our work emphasizes the role of vWAT in FA-related metabolic issues and suggests butyrate as a safe and promising adjunct for FA management.

## Highlights

• VWAT of FA mice shows altered expression of inflammatory and angiogenic genes
• FXN-lacking adipocytes rewire metabolism toward glycolysis and lactate production
• FA mice exhibit gut dysbiosis with a reduced abundance of butyrate-producing bacteria
• Dietary butyrate supplementation prevents vWAT alterations and glucose intolerance

## Graphical abstract

**Figure:**
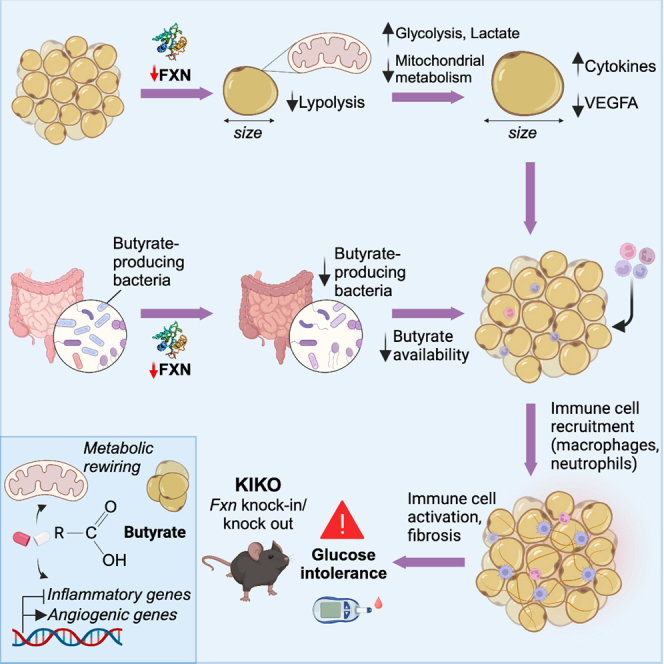

## Introduction

Friedreich’s ataxia (FA) is a rare neurodegenerative disease caused by the expansion of intronic trinucleotide repeat GAA from 8 to 33 repeats to >90 repeats in the *FXN* gene encoding frataxin protein (FXN). FXN resides in mitochondria and regulates mitochondrial iron transport and respiration.^1^ Apart from manifesting neurodegenerative signatures, FA patients are at higher risk to developing type 2 diabetes (T2D) and cardiomyopathy than general population, and these concur to aggravate the prognosis (extensively reviewed in the study by Tamarit et al.).^2^ It is now well ascertained that lipid metabolism is altered at systemic and cellular level in FA. FA patients show accumulation of lipid droplets (LD) in fibroblasts.^3^ LD accumulation has been also observed in FXN-deficient cultured rat cardiomyocytes and induced pluripotent stem cell-derived FA cardiomyocytes^4^^,^^5^ as well as in heart and brown adipose tissue of FA mouse models.^6^^,^^7^^,^^8^^,^^9^ Hepatic accumulation of fat (steatosis) in mice with a liver-specific FXN ablation^10^ and altered lipid metabolism associated with increased LD in glial cells of the drosophila FA model^11^ have been also observed.

White adipose tissue (WAT) is the tissue with the highest capacity to accumulate fats within LD and release them to other tissues in response to increased energetic demands. In addition to being a storage depot, WAT is a high active major endocrine organ impacting the metabolic function of several tissues and overall body metabolic homeostasis.^12^ Subcutaneous WAT is mainly involved in the buffering of circulating free fatty acids and triglycerides, thus exerting a protective function against systemic lipotoxicity and pathological accumulation of visceral WAT (vWAT).^13^^,^^14^ vWAT is present mainly in the abdominal region, and its expansion largely contributes to the onset of systemic low-grade inflammatory states that are at center stage of insulin resistance and T2D development.^15^ In addition to adipocytes, vWAT contains a great number of stromal vascular cells (SVCs) including endothelial cells, preadipocytes, and immune cells. Among the immune cells, macrophages play an important role in vWAT inflammation. Indeed, concomitant to pathological expansion, the ratio between the pro-inflammatory M1 macrophages and anti-inflammatory M2 macrophages increases, thus eliciting a major production of pro-inflammatory cytokines (e.g., tumor necrosis factor alpha (TNF-α)).^16^ Several evidence indicate that impaired oxidative function of mitochondria in vWAT adipocytes may be causally involved in its expansion and development of low-grade inflammation, insulin resistance, and T2D. Of note, the amphibolic organelles mitochondria take center stage in maintaining metabolic homeostasis in white adipocytes because of their involvement in fatty acid synthesis and esterification as well as lipid oxidation.^17^^,^^18^ Notably, increased visceral adiposity along with systemic chronic, low-grade inflammatory state was observed in FA patients.^19^^,^^20^ Also, obesogenic diet, which inevitably leads to WAT expansion, was demonstrated to exacerbate the metabolic dysfunctions caused by FXN deficiency in mice, indicating a role for FXN in the maintenance of WAT function.^21^

The recent approval of omaveloxolone for the treatment of FA underscores the progress made in addressing the disease.^22^^,^^23^ However, there is the continued need for research to develop other therapies to provide patients with safe and more diverse treatment options. Butyrate is a short-chain fatty acid, primarily produced through the fermentation of dietary fibers by beneficial bacteria in the gut. Supplementation with butyrate ameliorates body metabolism and increases insulin sensitivity.^24^^,^^25^^,^^26^ Moreover, butyrate ameliorates oxidative function of mitochondria and increases lipolysis in vWAT, thus limiting its expansion and restoring plasma leptin levels in diabetic mice.^27^^,^^28^ These findings highlight the potential of butyrate as a safe dietary supplement that could be utilized to counteract the T2D-like features in FA.

The FXN knockin/knockout (KIKO) mouse model, characterized by one allele of the *Fxn* (GAA)_230_ expansion mutation and one allele of the *Fxn* exon 4-deleted mutation,^29^ exhibits reduced FXN levels, subtle neurobehavioral symptoms, and well-detectable T2D-like metabolic abnormalities.^8^^,^^30^^,^^31^^,^^32^^,^^33^ This model provides valuable insights that can guide the development of therapeutic approaches targeting metabolic dysfunction in FA. In this study, we aimed to assess the potential dysfunction of vWAT in KIKO mice and explore butyrate supplementation as a safe option for addressing the T2D aspects of the disease.

## Results

### WAT of KIKO mice shows metabolic alterations and inflammatory hallmarks typical of type 2 diabetes

**Figure 1:**
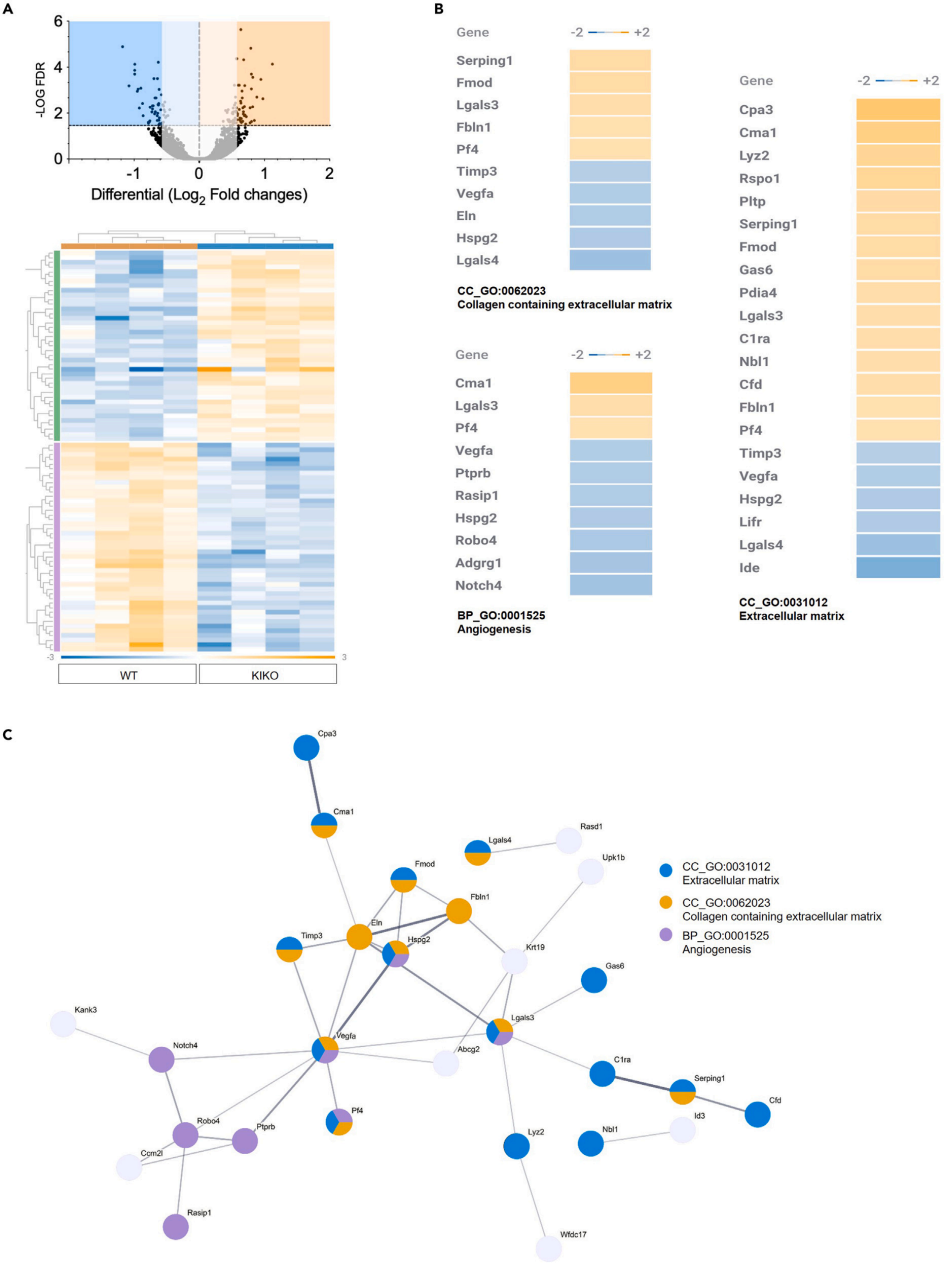
vWAT of KIKO mice shows alteration of gene expression (A) Volcano-plot representing differentially expressed genes between vWAT of 8-month-old WT and KIKO mice (upper panel). Heatmap representing the hierarchical clustering of significantly modulated genes (n = 4 male mice/group; fold change>1.5, <0.5; FDR<0.05) (bottom panel). (B) Functional enrichment analysis of modulated genes. (C) Protein-protein interaction network of modulated genes obtained by STRING platform. Nodes were colored according to the enriched GO term.

We previously showed that frataxin KIKO mice undergo weight gain starting at 8 months of age that was accompanied by an increase in circulating levels of leptin,^8^ suggesting that WAT dysfunction occurs in this mouse model. Hence, we performed bulk RNA sequencing (RNA-seq) analysis of the main WAT depot involved in T2D development i.e., vWAT. We found 97 differentially expressed genes (FC > 1.5; FC < 0.5; FDR<0.05; 41 up-, 56 downregulated) in vWAT of 8-month-old KIKO with respect to age-matched wild-type (WT) mice (Figure 1A). Functional enrichment analysis of the differentially expressed genes revealed angiogenesis and extracellular matrix as the modulated biological processes and cellular component, respectively (Figure 1B). Analysis of the cellular components also evidenced an enrichment of genes pertaining to collagen-containing extracellular matrix (Figure 1B). In the GO term extracellular matrix, the presence of genes typically expressed and secreted by macrophages and mast cells was found. These include i) the macrophage-specific Lyz2, involved in the onset of local WAT inflammation,^34^ and ii) the mast cell-specific carboxypeptidase A3 and chimerase 1, involved in the digestion of extracellular matrix and fibrosis development.^35^^,^^36^ Overall data indicated a vWAT rearrangement toward hypovascularization, inflammation, and fibrosis. To find a causal relationship between differentially expressed genes, we built a protein-protein interaction network using the free STRING platform (https://string-db.org/). The network depicted in Figure 1C highlights that some of the differentially expressed genes create an interconnected network with vascular endothelial growth factor A (VEGFA) representing a central hub. VEGFA is a growth factor inducing proliferation and migration of vascular endothelial cells and is essential for both physiological and pathological angiogenesis.

**Figure 2:**
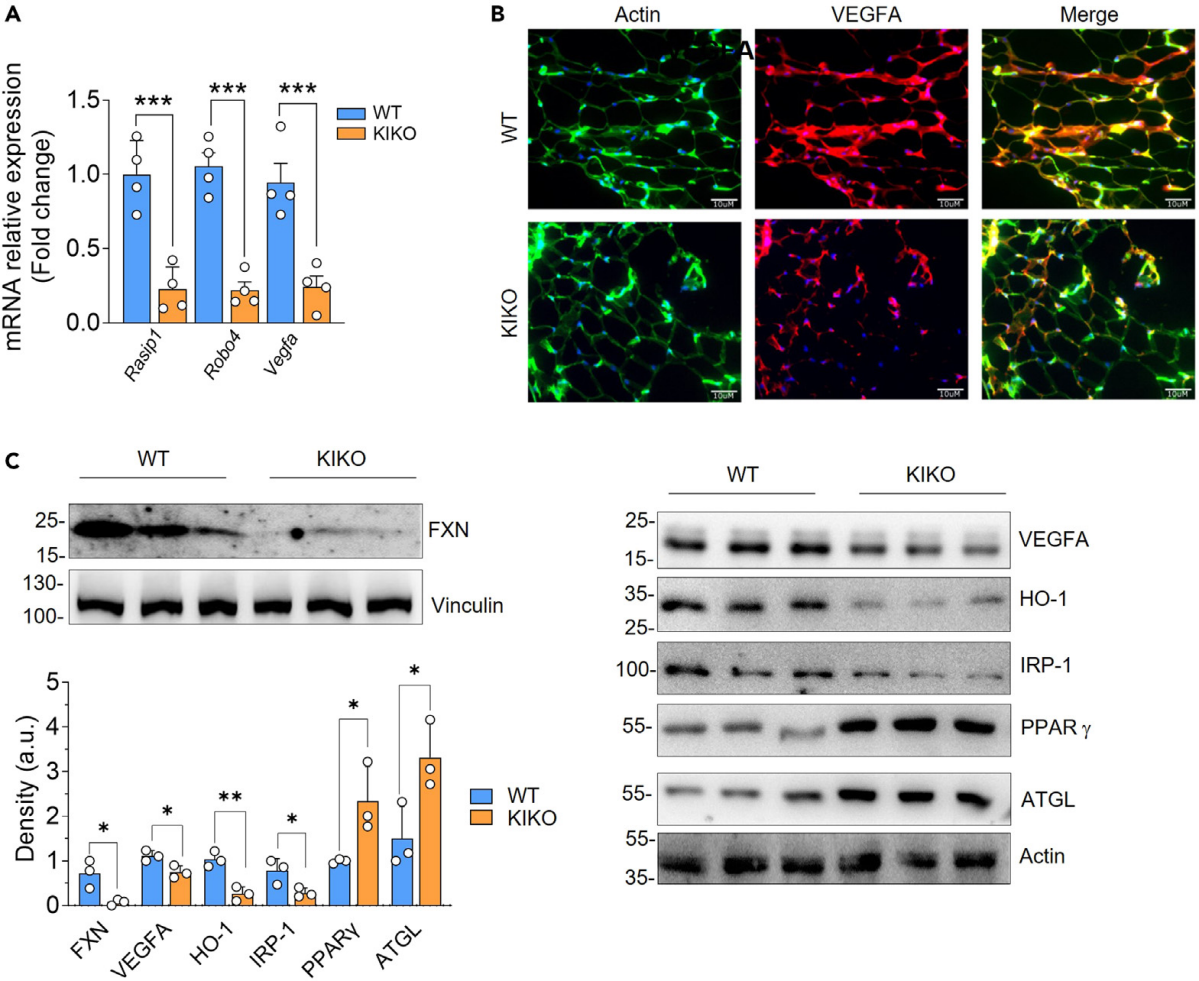
vWAT of KIKO mice shows alteration of angiogenesis and lipid metabolism (A) RT-qPCR analysis of angiogenic genes in 8-month-old WT and KIKO mice. Data are expressed as mean ± SD (n = 4 male mice/group; Student’s *t* test, ∗∗∗p < 0.001). (B) Representative immunofluorescence analysis of VEGFA (red). Phalloidin was used to stain F-actin (green) and Hoechst 33342 (blue) to visualize nuclei (Magnification 200×; scale bar 10 μm). (C) Western blot analysis of FXN and proteins related to blood vessel endothelial cell proliferation (VEGFA, HO-1, and IRP-1) and lipid metabolism (ATGL, PPARγ). Vinculin or actin was used as loading control. Density of immunoreactive bands was normalized with respect to loading control. Data are expressed as mean ± SD (n = 3 male mice/group; Student’s *t* test, ∗p < 0.05, ∗∗∗∗p < 0.0001).

RNA-seq data were validated through qPCR analysis. As reported in Figure 2A, *Vegfa* mRNA levels resulted in downregulation along with *Rasip1*—a protein essential for the correct assembly and angiogenic migration of endothelial cells^37^—and *Robo4*—an endothelial receptor that is involved in the maintenance of endothelial barrier organization and function.^38^ Immunofluorescence analysis and western blot analysis of VEGFA confirmed its downregulation in KIKO mice also at protein level (Figures 2B and 2C). Expectedly, FXN protein was significantly reduced in vWAT of KIKO mice (Figure 2C). This was accompanied by the decrease of the protein content of the downstream effectors of VEGFA, such as HO-1 and IRP-1, corroborating the overall downregulation of VEGFA pathway in vWAT of KIKO mice (Figure 2C). We previously demonstrated that FXN deficiency causes lipid accumulation in brown adipose tissue.^8^ Western blot analysis of hallmarks of lipid loading such as the adipose triglyceride lipase and the transcription factor and activator of lipid synthesis PPARγ indicated that lipid accumulation also occurs in vWAT (Figure 2C).

**Figure 3:**
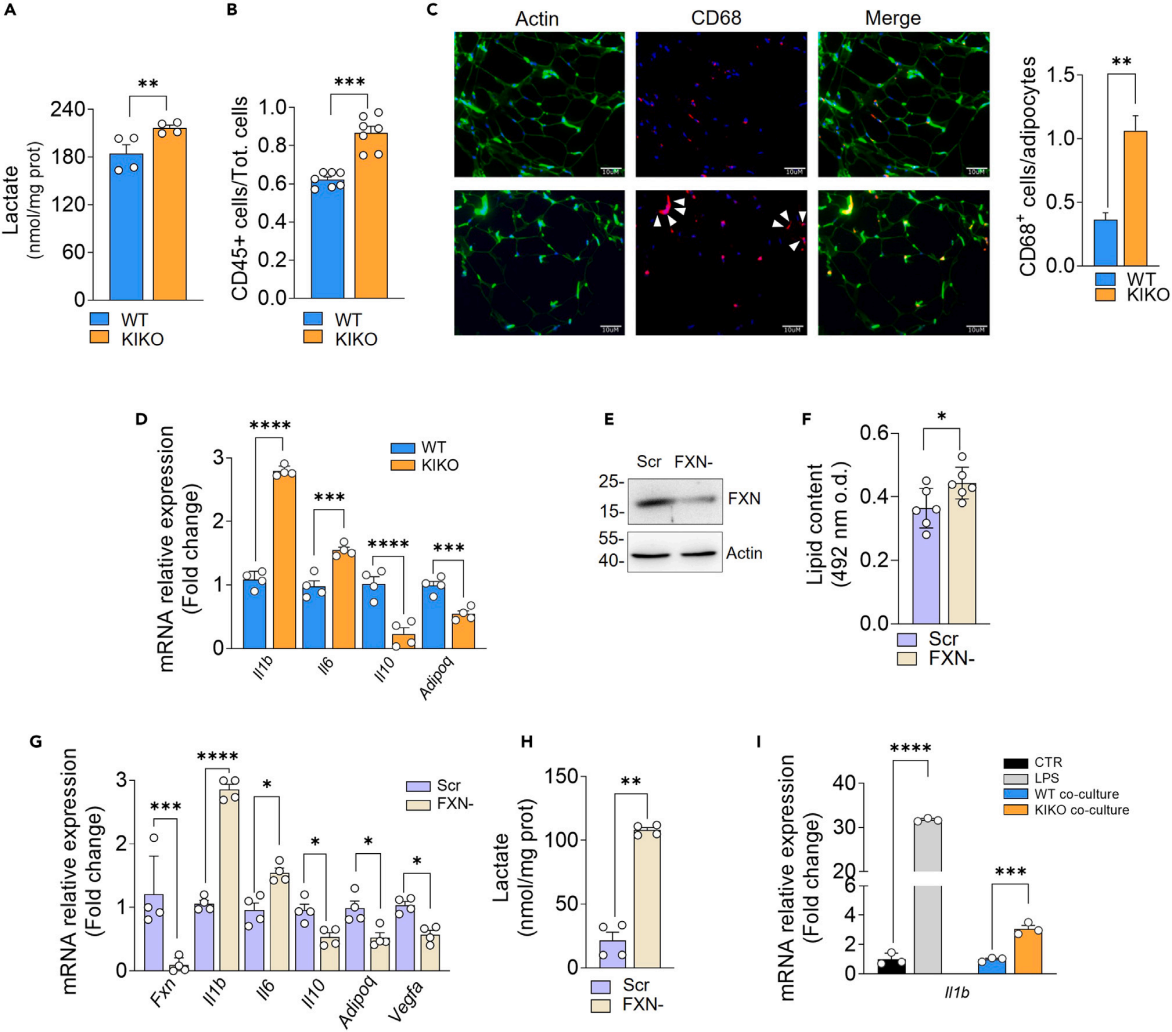
vWAT of KIKO mice shows signs of macrophage infiltration and inflammation (A) Spectrophotometric analysis of lactate released in culture medium of adipocytes differentiated from SVCs isolated from vWAT of 8-month-old WT and KIKO mice. Data are expressed as mean ± SD (n = 4 female mice/group; Student’s *t* test, ∗∗p < 0.01). (B) Immune cells (CD45^+^) in stromal vascular fractions isolated from vWAT of 8-month-old WT and KIKO mice by magnetic cell sorting. Data are expressed as mean ± SD (n = 7 mice/group; 3 male, 4 female; Student’s *t* test, ∗∗∗∗p < 0.001). (C) Representative immunofluorescence analysis of CD68^+^ M1 macrophage infiltrates (red) in vWAT of 8-month-old WT and KIKO mice. Phalloidin was used to stain F-actin (green) and Hoechst 33342 (blue) to visualize nuclei (Magnification 200×; scale bar 10 μm). White arrows indicate the presence of crown-like structures around adipocytes. Quantification of macrophage number per adipocytes is reported (*right panel*). Data are expressed as mean ± SD (n = 4 male mice/group; Student’s *t* test, ∗∗p < 0.01). (D) RT-qPCR analysis of inflammatory genes in vWAT of 8-month-old WT and KIKO mice. Data are expressed as mean ± SD (n = 4 male mice/group; Student’s *t* test, ∗∗∗p < 0.001, ∗∗∗∗p < 0.0001). (E–H) Murine 3T3-L1 adipocytes were transfected with a shRNA against FXN (FXN-) or with a Scr shRNA. Western blotting analysis of FXN and actin (loading control). Immunoblot reported is representative of three giving similar results (E). Intracellular lipid content determined by spectrophotometric measurement of eluted oil red O. Data are expressed mean ± SD (n = 6 biological replicates; Student’s *t* test, ∗p < 0.05) (F). qPCR analysis of *Fxn,* inflammatory *(Il1b, Il6, Il10, Adipoq)*, and *Vegfa* mRNAs. Data are expressed as mean ± SD (n = 4 biological replicates; Student’s *t* test, ∗p < 0.05, ∗∗∗p < 0.0001, ∗∗∗∗p < 0.0001) (G). Spectrophotometric analysis of lactate released in culture medium. Data are expressed as mean ± SD (n = 4 biological replicates; Student’s *t* test, ∗∗p < 0.01) (H). (I) qPCR analysis of *Il1b* mRNA in RAW264.7 macrophages co-cultured with adipocytes differentiated form SVCs isolated from vWAT of 8-month-old female WT or KIKO mice. LPS treatment was used as positive control. Data are expressed as mean ± SD (n = 3 biological replicates; Student’s *t* test, ∗∗∗p < 0.001, ∗∗∗∗p < 0.0001).

Hypovascularization and vWAT expansion can lead to lowering of oxygen availability, decrease of mitochondrial respiration, and metabolic reprogramming toward glycolysis and lactate production. Notably, increased levels of lactate in WAT are typical of T2D conditions.^39^^,^^40^ In order to pinpoint possible metabolic alterations of WAT, we isolated SVCs, and preadipocytes were induced to differentiate. Analysis of lactate concentration in culture medium showed that mature adipocytes of KIKO mice had a higher rate of lactate production than WT mice (Figure 3A). Lactate was previously regarded as the waste product of glycolysis. Recently, it has emerged that lactate serves as a danger signal that promotes polarization of resident macrophages toward a pro-inflammatory M1-like state in the context of obesity.^39^^,^^40^ Based on this evidence and our RNA-seq results, we determined the leukocyte abundance in SVCs isolated from vWAT. After isolation of CD45^+^ cells by magnetic cell sorting, we found that leukocyte content was increased in vWAT of KIKO mice (Figure 3B). Next, we evaluated the presence of macrophage infiltrates by immunofluorescence analyses of vWAT sections. We used differentiation cluster 68 (CD68) as a marker for M1-like macrophages. vWAT of KIKO mice displayed a higher number of CD68^+^ cells per adipocytes when compared to WAT of WT mice (Figure 3C). Moreover, some crown-like structures (consisting in several macrophages surrounding a single adipocyte) typical of inflamed WAT were observable in KIKO mice (Figure 3C). Thus, we investigated mRNA level of key factors involved in the regulation of the inflammatory response. vWAT of KIKO mice showed a higher expression of the pro-inflammatory *Il1b* and *Il6* genes compared to WT mice. Consistent with the hypothesis of increased inflammatory processes within vWAT of KIKO mice, anti-inflammatory *Il10* was found downregulated (Figure 3D). These data were confirmed by analyzing the expression of *Adipoq* gene encoding for adiponectin, an anti-inflammatory and antidiabetic hormone secreted by adipocytes.^41^^,^^42^ Adiponectin improves insulin sensitivity, inhibits macrophage-mediated inflammation, and is downregulated in T2D patients.^43^ In line with the observed T2D signatures, *Adipoq* mRNA expression result significantly decreased in vWAT of KIKO mice (Figure 3D).

To better elucidate the role of adipocytes in the observed macrophage recruitment and vWAT inflammation, we downregulated FXN in a cellular model of murine (3T3-L1) white adipocytes. In line with the results obtained in vWAT, FXN deficiency caused lipid accumulation (Figures 3E–3G), VEGFA and inflammatory marker alteration (Figure 3G), and extracellular lactate increase (Figure 3H). Interestingly, in co-culturing conditions, KIKO-derived adipocytes but not WT-derived adipocytes were able to induce *Il1b* gene expression in RAW264.7 macrophages (Figure 3I), pointing to a central role of adipocytes in triggering macrophage activation upon FXN deficiency.

### Dietary butyrate supplementation improves systemic and vWAT metabolism in KIKO mice

**Figure 4:**
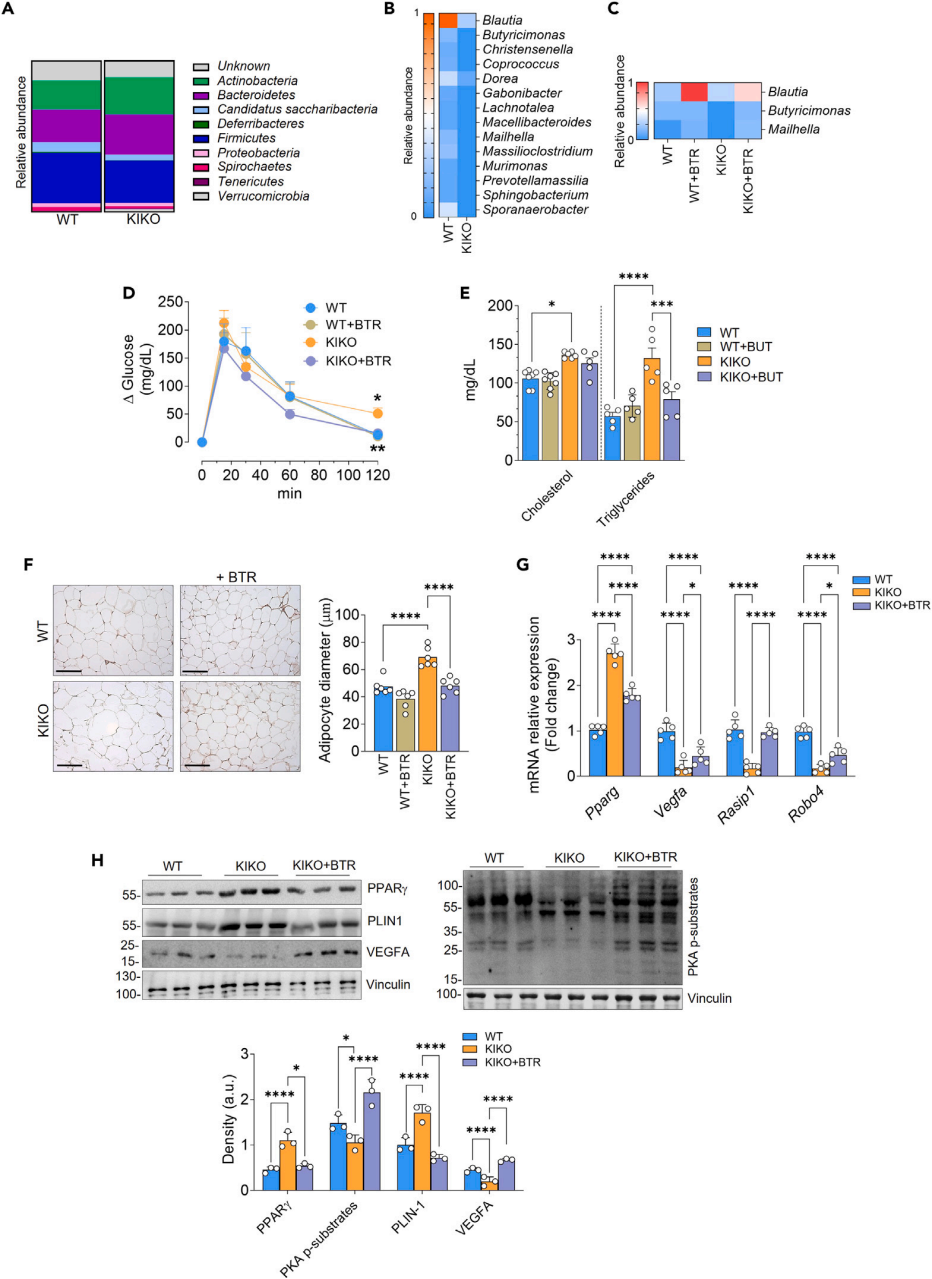
Butyrate supplementation counteracts development of T2D-like symptoms in KIKO mice Four-month-old male WT and KIKO mice were fed with normal diet or with diet supplemented with butyrate (+BTR) for 16 weeks up to 8 months of age. (A and B) Relative abundance of microbiota phyla (SILVA database analysis) (A) and heatmap representation of butyrate-producing bacteria (genera in terms of relative abundance) (B) in fecal samples of 8-month-old WT and KIKO mice (n = 12 male mice/group). (C–H) Four-month-old male WT and KIKO mice were fed with normal diet or with diet supplemented with butyrate (+BTR) for 16 weeks up to 8 months of age. Heatmap representation of butyrate-producing bacteria in fecal samples whose relative abundance is modulated by butyrate in KIKO mice (n = 6 male mice/group) (C). Oral glucose tolerance test (OGTT) was carried out after oral administration of 2 g of dextrose/kg body mass. The data are presented as Δglucose, which was calculated by subtracting the glucose concentrations in blood at the starting point (point 0) from the concentrations measured at subsequent time points (20, 30, 60, 120 min) following oral glucose administration. Data are expressed as mean ± SD (n = 6 male mice/group; ANOVA test, ∗p < 0.05 vs. WT mice; ∗∗p < 0.01 vs. butyrate-untreated KIKO mice) (D). Plasma triglycerides and cholesterol levels. Data are expressed as mean ± SD (at least n = 5 male mice/group; ANOVA test, ∗p < 0.01, ∗∗∗p < 0.001, ∗∗∗∗p < 0.001) (E). Representative histology images of vWAT from 4-month-old WT and KIKO mice fed with normal diet or treated with butyrate (+BTR) for 16 weeks up to 8 months of age after staining with H&E (F, left panel). Adipocyte size was represented as lipid droplet diameters (F, right panel). Data are expressed as mean ± SD (n = 6 male mice/group, ANOVA test, ∗∗∗∗p < 0.0001). RT-qPCR analysis of *Pparg* and angiogenic mRNAs. Data are expressed as mean ± SD (n = 5 male mice/group; ANOVA test, ∗p < 0.05; ∗∗p < 0.01; ∗∗∗∗p < 0.0001) (G). Western blot analysis of proteins involved in lipid metabolism (PPARγ, PLIN1, phospho-PKA substrates) and VEGFA. Vinculin was used as loading control. Density of immunoreactive bands was normalized with respect to related loading control. Data are expressed as mean ± SD (n = 3 male mice/group; ANOVA test, ∗p < 0.05, ∗∗∗∗p < 0.001) (H). See also Figure S1.

Among the typical events associated with low-grade inflammation, altered gut microbiota is also included.^44^ Importantly, a very strict cross talk exists between WAT and gut microbiota that synergistically contributes to maintaining body metabolic homeostasis.^45^^,^^46^ Metagenomic analysis of fecal samples showed an altered microbiota composition in KIKO mice (Figure 4A). Deeper analysis conducted at genus level highlighted an almost total absence of butyrate-producing bacteria in gut microbiota of KIKO mice (Figure 4B). Butyrate is predominantly produced by gut microbes, and it is now ascertained that the decrease of butyrate-producing bacteria inevitably leads to diminution of systemic butyrate availability.^47^ Prompted by this, we proceeded to evaluate the potential of butyrate supplementation in limiting vWAT alterations and exerting antidiabetic effects. We supplemented mice with sodium butyrate by adding it in food pellets (5 g · kg−1 · day−1 at the normal daily rate of calorie intake) starting at 4 months of age and continuing for 16 weeks until 8 months of age, time in which KIKO mice manifest metabolic alterations and weight gain.^8^ The decision to use this plan of butyrate supplementation was based on previous research demonstrating the safety and beneficial antidiabetic effects of butyrate at this dose and administration schedule.^25^ Accordingly, we did not observe any alterations in food and water intake upon butyrate treatment (Figures S1A and S1B). Furthermore, the canonical bioclinical markers of tissue functions (i.e., GOT, GPT, LDH, creatinine) revealed no significant differences between the butyrate-treated groups and the control groups (Figure S1C), confirming its well-tolerated nature.

Metagenomic analysis revealed that butyrate supplementation restored the abundance of certain butyrate-producing bacteria in fecal samples from both WT and KIKO mice, with KIKO mice showing levels comparable to or even larger than those of untreated WT mice (Figure 4C). These data stimulated us to evaluate whether butyrate treatment was able to mitigate systemic metabolic alterations in KIKO mice. The oral glucose tolerance test revealed that butyrate supplementation was effective in restraining glucose intolerance in KIKO mice, as a significant recovery of normal glycemia levels was observed after 120 min from glucose administration (Figure 4D). Regarding lipidemia, we found that butyrate was effective in buffering hypertriglyceridemia but not total cholesterol levels, even though a tendency was noted (Figure 4E).

Histochemical evaluation displayed an increase in adipocyte size in vWAT of KIKO compared to WT mice, confirming vWAT expansion (Figure 4F). Notably, butyrate treatment was able to reduce the diameter of adipocytes in vWAT of KIKO mice, with vWAT adipocytes of butyrate-treated KIKO mice reaching a size comparable to that observed in vWAT adipocytes of untreated WT mice (Figure 4F). These results led us to evaluate whether vWAT dysfunction in KIKO mice could be recovered by butyrate treatment. Western blot and qPCR analyses showed that markers of lipid accumulation (PPARγ, PLIN-1) and hypovascularization (VEGFA, Rasip, Roboa4) came back to control values upon butyrate treatment (Figures 4G and 4H). Accumulation of lipids depends on increased PPARγ-mediated lipogenesis and inhibition of the hormone-sensitive lipolytic cascade activated by protein kinase A (PKA).^12^ By western blot analysis, we observed a decrease of the level of PKA-phosphosubstrates, and butyrate was able to revert this event (Figure 4H). Overall, these results underline the beneficial effects of butyrate in maintaining lipid homeostasis and vascularization in vWAT of KIKO mice.

### Dietary butyrate supplementation restrains vWAT inflammation in KIKO mice

**Figure 5:**
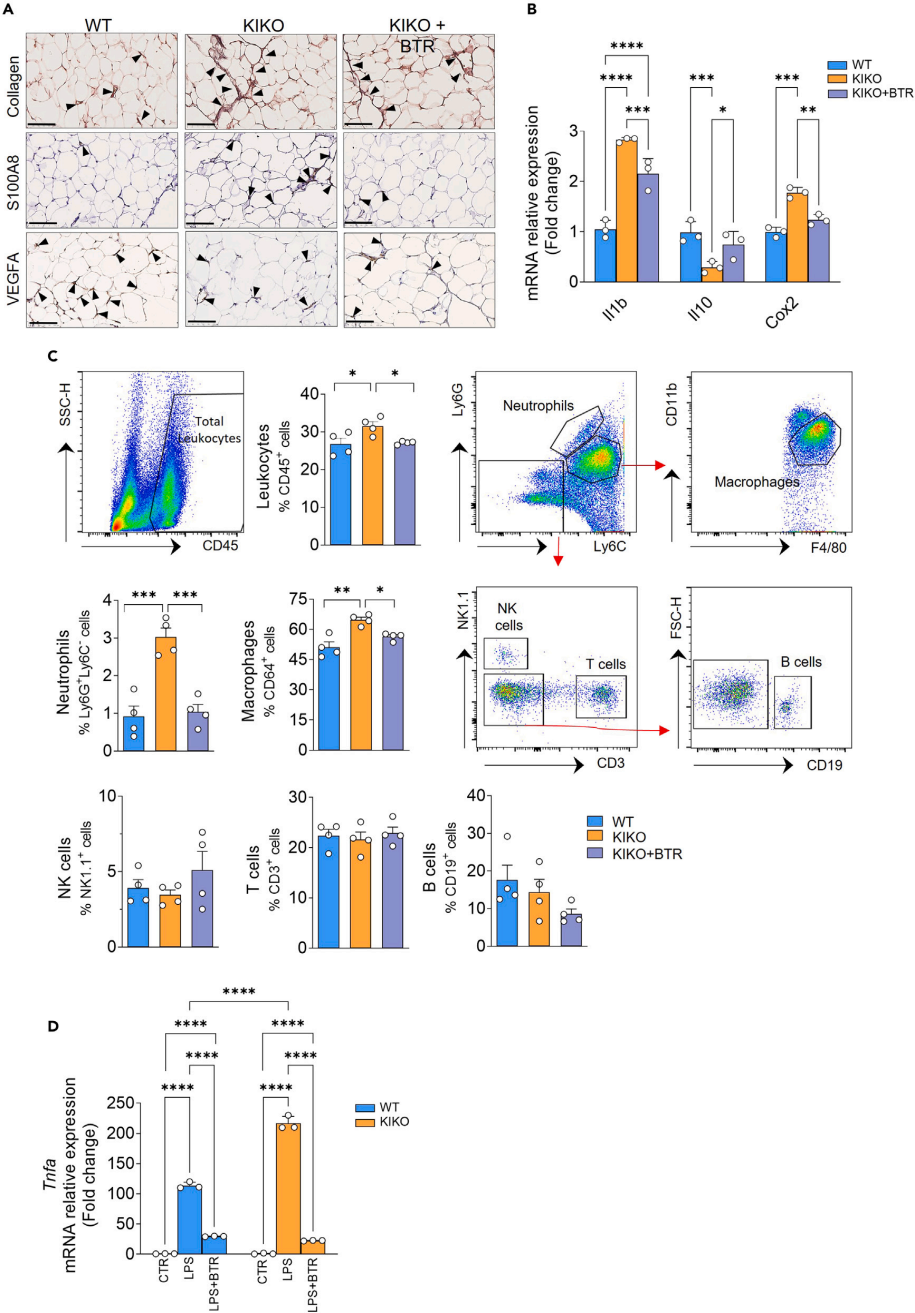
Butyrate treatment prevents the onset of inflammation in vWAT of KIKO mice Four-month-old male WT and KIKO mice were fed with normal diet or with diet supplemented with butyrate (+BTR) for 16 weeks up to 8 months of age. (A) Representative histology images of vWAT to detect collagen (Masson trichrome staining), VEGFA (staining with VEGFA antibody), or immune cell infiltrates (staining with S100a8 antibody). Arrowheads indicate fibrotic septa, VEGFA positive staining, or inflammatory infiltrates with scattered cells (Magnification 200×; scale bar 10 μm). (B) RT-qPCR analysis of inflammatory genes. Data are expressed as mean ± SD (n = 3 male mice/group; ANOVA test, ∗p < 0.05, ∗∗p < 0.01, ∗∗∗p < 0.001, ∗∗∗∗p < 0.0001). (C) SVCs were isolated from vWAT and analyzed through high-dimensional flow cytometry by using specific antibodies to detect total leukocytes, neutrophils, macrophages, T cells, NK cells, and B cells. Gating strategies to detect the immune cell subpopulations are illustrated. Data are expressed as mean percentage of positive cells ±SD (n = 4 male mice/group; ANOVA test, ∗p < 0.05, ∗∗p < 0.01, ∗∗∗p < 0.001). (D) RT-qPCR analysis of *Tnfa* mRNA in BMDM isolated from 8-month-old female WT and KIKO mice, and treated with LPS (500 ng/mL, 16 h) alone or in combination with butyrate (500 μM). Data are expressed as mean SD (n = 3 biological replicates; ANOVA test, ∗∗∗∗p < 0.0001). See also Figure S1.

Immunohistochemical analyses also proved that, along with the recovery of VEGFA levels, collagen content and immune cell infiltrates were reduced upon butyrate treatment (Figure 5A; Figures S1D–S1F). In parallel, butyrate restrained the upregulation of the pro-inflammatory *Il1b* and *Cox*2 genes and restored the mRNA level of the anti-inflammatory cytokine *Il10* (Figure 5B). Accordingly, we sought to deeply characterize innate and adaptive immune cell dynamics by investigating potential recruitment of other immune cell populations in vWAT and whether butyrate was able to exert an impact on this. To this end, we isolated SVCs of vWAT, and the single-cell suspension was analyzed by high-dimensional flow cytometry. As expected, a higher percentage of total CD45^+^ leukocytes were observed in SVCs of KIKO compared to WT mice (Figure 5C). We then applied a consequential gating strategy to identify the percentages of the different cell subsets of leukocytes, i.e., macrophages (CD11b^+^F4/80^+^CD64^+^ cells), neutrophils (Ly6G^+^ Ly6C^−^ cells), T cells (CD3^+^ cells), natural killer (NK) (NK1^+^ cells), and B cells (CD19^+^ cells). Interestingly, vWAT of KIKO mice showed a higher percentage of macrophages and neutrophils compared to WT mice (Figure 5C), corroborating our previous findings by immunohistochemical analyses (Figures 3C and 5A). By contrast, percentages of T and B lymphocytes as well as NK cells were not changed (Figure 5C), suggesting that NK cells and cells of the adaptive immunity do not contribute to the inflammation of vWAT in KIKO mice. Notably, butyrate was able to prevent leukocyte infiltration, and this was due to reduction of both macrophages and neutrophils (Figure 5C). Immune cells have higher capacity to produce inflammatory cytokines than adipocytes; hence, once recruited in vWAT by adipocyte-derived signals, immune cells could enhance the production of inflammatory mediators. Hence, we questioned whether FXN deficiency could also influence the inflammatory response in immune cells and butyrate to restrain this event.

To this end, we isolated bone-marrow-derived macrophages (BMDMs) from WT and KIKO mice. BMDMs were treated with lipopolysaccharide (LPS) to reproduce an inflammatory insult. Upregulation of the expression of the *Tnfa* gene was obtained after LPS stimulation in both WT and KIKO BMDMs (Figure 5D); however, even though FXN deficiency did not influence basal *Tnfa* expression, upon LPS treatment, *Tnfa* upregulation was more marked in KIKO than in WT cells. Notably, butyrate was able to significantly buffer the LPS-mediated inflammatory challenge (Figure 5D), suggesting that this molecule has an anti-inflammatory action also in myeloid cells and likely at systemic level.

### Butyrate supplementation ameliorates adipocyte metabolism in KIKO mice

**Figure 6:**
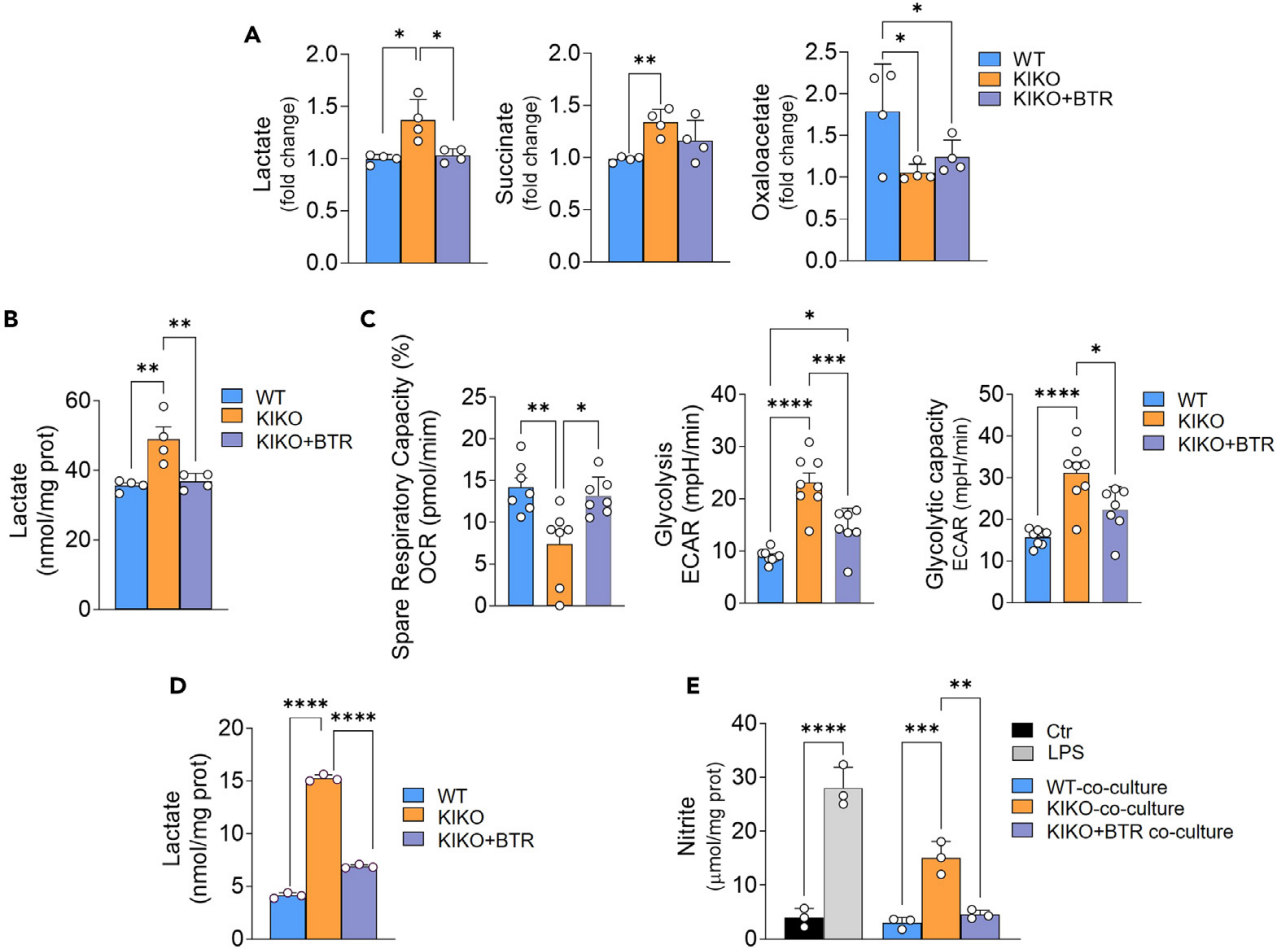
Butyrate treatment improves adipocyte metabolism in KIKO mice Four-month-old male WT and KIKO mice were fed with normal diet or with diet supplemented with butyrate (+BTR) for 16 weeks up to 8 months of age. (A) Targeted metabolomic analysis of vWAT. Data are expressed as mean ± SD (n = 4 male mice/group; ANOVA test, ∗p < 0.05, ∗∗p < 0.01). (B) Spectrophotometric analysis of lactate released in culture medium of vWAT SVCs. Data are expressed as mean ± SD (n = 4 male mice/group; ANOVA test, ∗∗p < 0.01). (C) Real-time measurement of spare Respiratory capacity, glycolysis, and glycolytic capacity in vWAT SVCs. ECAR: Extracellular Acidification Capacity. Data are expressed as mean ± SD (n = 4 male and n = 3 female mice each group; ANOVA test, ∗p < 0.05; ∗∗p < 0.01; ∗∗∗p < 0.001; ∗∗∗∗p < 0.0001). (D) Spectrophotometric analysis of lactate released in culture medium of differentiated adipocytes from vWAT of 8-month-old WT and KIKO mice and treated with butyrate (500 μM, 16 h). Data are expressed as mean ± SD (n = 3 female; ANOVA test, ∗∗∗∗p < 0.00001). (E) Nitrite concentration in culture medium of RAW264.7 macrophages co-cultured with adipocytes isolated from 8-month-old WT or KIKO female mice and treated with BTR (500 μM, 16 h). LPS treatment (500 ng/mL, 16 h) of RAW264.7 macrophages was used as positive control. Data are expressed as mean ± SD (n = 3 biological replicates; ANOVA test, ∗∗∗p < 0.001, ∗∗∗∗p < 0.0001). See also Table S1.

To have a comprehensive view of the effects of butyrate on WAT metabolism, we then performed targeted metabolomic analyses of several metabolites (about 100) pertaining to glycolysis, pentose phosphate pathway, urea and Krebs cycle, and other metabolites such as carnitines, nucleotides, amino acids, and catecholamines. Only few metabolites were significantly affected in vWAT of KIKO mice (i.e., lactate, oxaloacetate, and succinate) (Figure 6A). Among these, only lactate underwent significant reduction upon butyrate treatment. We then performed XF Seahorse real-time monitoring of cell metabolism in SVCs isolated from vWAT of KIKO mice to understand whether lactate hyperproduction observed with metabolomic analyses was associated with augmented glycolytic rate, and whether butyrate treatment was able to mitigate such phenomenon *in vivo*. Lactate production of KIKO SVCs was attenuated following 16 weeks treatment with butyrate (Figure 6B). Expectedly, in SVCs of KIKO mice, we found a significant decrease of spare respiratory capacity, which is a measure of the ability of mitochondria to respond to increased energy demand (Figure 6C). The monitoring of extracellular acidification rate, after addition of saturating amount of glucose, revealed that KIKO SVCs have higher glycolytic rate than WT adipocytes (Figure 6C). The addition of the mitochondrial respiration inhibitor oligomycin revealed that also the maximum glycolytic capacity was higher in KIKO than WT adipocytes (Figure 6C). Notably, butyrate was able to recover mitochondrial respiration capacity and to reduce glycolytic metabolism in KIKO SVCs (Figure 6C). We also tested the effects of butyrate on lactate production of SVCs after induction of adipocyte differentiation. As reported in Figure 6D, treatment with butyrate lowered the concentration of lactate in culture medium of KIKO adipocytes. These results point to the ability of the *in vivo* treatment with butyrate to shift cellular metabolism from glycolysis to mitochondrial respiration, thus avoiding the release of anti-lipolytic and pro-inflammatory lactate and cytokines in WAT.

## Discussion

In this study, we demonstrated that FXN deficiency leads to vWAT dysfunction, which consists of the expansion of adipocyte size, hypovascularization, production of pro-inflammatory adipokines, immune cell recruitment, and fibrosis. These findings recapitulate what is observed under T2D conditions in vWAT.

We previously demonstrated that FXN deficiency causes lipid accumulation and lower thermogenic capacity in brown adipocytes,^8^ indicating a possible involvement of decreased antidiabetic activity of brown adipose tissue (BAT) in the onset of metabolic complications observed in FA patients. As in BAT, in vWAT of our FA mouse model, we found altered lipid metabolism due to increased expression of lipogenic markers and impaired lipolytic activity. It appears that vWAT is more affected than BAT in FA mice; inasmuch as besides fat accumulation, we found altered expression of genes related to angiogenesis, including VEGFA, the master regulator of this process also in vWAT.^48^^,^^49^ Insufficient angiogenic potential is a dominant contributor to the dysfunctional WAT, as hypoxic condition triggers chronic low-grade inflammation predominantly characterized by pro-inflammatory macrophage infiltration.^48^ Intriguingly, VEGFA also exerts an antidiabetic action by functioning as a promoter of insulin sensitivity and as an anti-inflammatory M2 macrophage attractant in WAT,^50^ suggesting that the VEGFA downregulation observed in vWAT may participate in the development of insulin resistance and inflammation.

We demonstrated that FXN-deficient white adipocytes are *per se* more prone to produce pro-inflammatory cytokines than normal adipocytes, arguing that they can play an active role in macrophage recruitment and activation. In vWAT of KIKO mice, we also disclosed high degree of immune cell infiltrates, mostly macrophages and neutrophils, in association with the upregulation of fibrosis markers (e.g., collagen) and pro-inflammatory cytokine production. These findings recapitulate the fibrotic inflammatory phenotype typical of vWAT in T2D.^49^^,^^51^

Enlarged vWAT can release hormones and other substances that may contribute to insulin resistance, cardiomyopathy, and low-grade inflammation.^52^^,^^53^ Apart from T2D and cardiomyopathy, signs of low-grade inflammation with high levels of inflammatory cytokine expression in circulating leukocytes were found in FA patients.^20^ Additionally, FXN downregulation enhanced inflammatory response in macrophages, indicating that a positive loop of inflammation occurs in vWAT of FXN-deficient mice, with adipocytes favoring the infiltration of immune cells that are more responsive to inflammatory stimuli. This evidence and our results point to the role of FXN in the maintenance of overall body immune homeostasis.

Augmented lactate levels in adipose tissue and plasma are widely reported in obese/diabetic subjects.^39^^,^^54^ Metabolically, we found that FXN-deficient adipocytes have increased glycolytic activity and consequent hyperproduction of lactate. The switch toward glycolytic metabolism may originate from defective mitochondrial respiration and/or scarce oxygen availability due to reduced angiogenesis and vascularization. Lactate is a redox molecule that, in adipose tissues, displays a wide range of biological effects both through its binding to membrane receptors and its transport and subsequent effect on intracellular metabolism.^55^ Lactate produced by adipocytes inhibits lipolysis in an autocrine/paracrine manner through inhibition of PKA.^56^ Accordingly, we observed a decrease of PKA activity in vWAT of KIKO mice. High lactate levels increase cardiovascular risk^54^^,^^57^^,^^58^ and promote macrophage recruitment and WAT inflammation.^39^ We found that co-culturing macrophages with FXN-deficient adipocytes promotes a more efficient inflammatory response. This result suggests that, apart from the increased production of inflammatory cytokines, FXN-deficient adipocytes produce higher level of lactate that could be responsible for macrophage activation.

In FA patients, body weight and body mass composition can vary among individuals.^59^^,^^60^^,^^61^^,^^62^ Some FA individuals with insulin resistance exhibit increased body weight and altered adipose tissue distribution, with increased fat deposition in the visceral region.^60^ Other FA patients have a lower body weight compared to healthy individuals, which could be attributed to loss of lean mass due to muscle wasting.^61^^,^^62^^,^^63^ These changes in body weight and composition highlight the complex metabolic implications of FA and the need for comprehensive studies to better understand their underlying mechanisms and potential impact on the disease.

In mouse models of FA, phenotypic outcomes can vary based on specific genetic modifications, tissue-specific FXN deletion, and other introduced factors, recapitulating what described previously in FA patients. What observed in this work on KIKO mice adheres with the phenotype observed in FA patients with increased body weight, visceral fat, and insulin resistance.^60^ By contrast, recently developed FA models such as UCLA and YG8-800 mice, featuring inducible FXN knockdown and larger triplets’ expansion, exhibit early onset, severe neurodegeneration, and cardiomyopathy, but do not report T2D-like signs and experience weight loss.^64^ The conditional MCK FXN knockout mouse, targeting cardiac and skeletal muscle, develops pronounced cardiomyopathy and significant weight loss,^65^ which is likely attributed to the decrease in lean mass due to cardiomyopathy/skeletal muscle dysfunction rather than a decrease in fat mass.

In line with KIKO mice, other models characterized by late-onset and mild neurological defects, such as YG8R and YG22R, have reported weight gain and certain aspects of T2D.^66^ Based on our findings, it is reasonable to propose that these animals may experience expansion and inflammation of WAT. Considering the complex metabolic implications observed in FA patients, the mild and slowly progressing neurological phenotype of the KIKO model allows to specifically investigate the metabolic aspects of FA without the confounding effects of severe neurodegeneration and cardiomyopathy. This focus on the metabolic manifestations of the disease provides valuable insights into the contribution of WAT dysfunction to the overall pathology of FA. Mouse models with mild neurological defects and cardiomyopathy, but also featuring increased body weight and visceral adiposity as KIKO mouse, emerge as more suitable models for understanding potential therapeutic targets in a significant subset of FA patients with these specific characteristics. This is particularly relevant given the adverse impact of T2D on the prognosis of FA patients.

Dysbiosis has been linked to T2D and various neurological disorders such as multiple sclerosis and autism spectrum disorders.^67^^,^^68^^,^^69^ A very strict cross talk exists between vWAT and gut microbiota that synergistically contribute to maintaining body metabolic homeostasis. Indeed, altered gut microbial ecosystems have been associated with vWAT dysfunction (i.e., expansion, inflammation, and insulin resistance), low-grade chronic inflammation, and systemic metabolic perturbations, including T2D.^70^ A decrease in butyrate-producing bacteria has been causally involved in vWAT expansion and inflammation, as well as in T2D development.^71^^,^^72^ To our knowledge, no attempts have been made to unravel whether the microbiota is altered in FA. Herein, we show that our FA mouse model has an altered gut microbiota composition compared to healthy mice, with a reduction in the abundance of butyrate-producing bacteria. The causes of such reduction have not been explored in the present work and the possible occurrence of gut inflammation deserves further and deeper investigation. Mitochondrial metabolism of colonocytes, by consuming O_2_, maintains the predominance of anaerobic bacteria in the gut, including butyrate-producing bacteria.^73^ It can be argued that mitochondrial dysfunction, which is likely to occur in FXN-deficient colonocytes, increases oxygen availability and the proliferation of facultative anaerobic bacteria, while reducing the abundance of butyrate-producing bacteria (dysbiosis). This dysbiosis could contribute to the establishment of a T2D-like inflammatory state in vWAT.

While further research is needed to fully understand the role of the microbiota in FA, our findings suggest that alterations in the microbiota may be involved in the pathophysiology of the disease and provide a potential target for therapeutic interventions. Strategies that target the gut microbiota, such as butyrate supplementation, probiotics, or prebiotics, may have potential for improving symptoms and disease progression in FA.

The decrease in butyrate-producing bacteria strongly indicates that systemic butyrate availability could be reduced in KIKO mice. Butyrate has been studied for its potential anti-inflammatory and neuroprotective effects, as well as its role in diabetes management.^74^^,^^75^ Our data demonstrate that butyrate supplementation may be effective in counteracting vWAT dysfunction and T2D-like symptoms in the context of FA. Specifically, at the metabolic level and similarly to what was previously described in colonocytes,^76^ butyrate increased respiratory capacity of mitochondria with FXN deficiency in vWAT, while decreasing glycolytic activity and lactate production. In parallel, butyrate hindered the accumulation of fats, and this was accompanied by the maintenance of angiogenic VEGFA levels to levels comparable to those of healthy mice. As also described in other models of T2D,^77^^,^^78^ butyrate supplementation ameliorated glycemic profile in KIKO mice. Regarding inflammatory signatures, butyrate reduced leukocyte infiltration (i.e., macrophages, neutrophils), and production of pro-inflammatory cytokines.

Butyrate has a wide range of pleiotropic effects and mechanisms of action. Although the precise mechanisms by which butyrate acts in our FA models have not been deeply investigated in this study, it is likely that the beneficial effects of butyrate can be mediated by the inhibition of histone deacetylases, thus epigenetically modulating the expression of genes involved in inflammation and energy metabolism.^79^ Butyrate can also have a direct action on mitochondria. For instance, being a short-chain fatty acid, butyrate can be directly funneled into mitochondria and enhance mitochondrial respiration and fatty acid oxidation and impede lipid accumulation.^76^ It can be hypothesized that the observed decrease in glycolysis and subsequent recovery of mitochondrial respiratory capacity in FXN-deficient adipocytes may be due to the redirection of reducing equivalents from butyrate β-oxidation toward succinate dehydrogenase (CII activity). Notably, measurement of CII activity in KIKO mice revealed a partial reduction of approximately 40%, compared to controls.^31^ A complete abrogation of complex II activity in both patients and experimental models of FA has not been reported.^31^^,^^80^ Notably, a compensatory activation of CII has even been found in cerebellar neurons of the YG8R mouse model.^81^ These findings lend support to the broad applicability of butyrate for restoring mitochondrial respiration upon FXN deficiency.

We cannot exclude that the beneficial effects of butyrate supplementation could be also dependent on the recovery of gut butyrate-producing bacteria as disclosed in FA mice. This result aligns with existing evidence that exogenous butyrate has the potential to improve gut microbiota dysbiosis in animal models of obesity with high-fat diet or systemic lupus erythematosus. This improvement is achieved by increasing the abundance of butyrate-producing bacteria.^82^^,^^83^ It can be hypothesized that the presence of exogenous butyrate may impact the competitive interactions among different microbial species by regulating the pH of the gut lumen.^84^ By maintaining a mildly acidic pH, butyrate may create a favorable environment for butyrate-producing bacteria, enabling them to outcompete with other bacteria.^85^

Overall, our results suggest that vWAT is dysfunctional and microbiota altered in FA. Butyrate supplementation prevents vWAT expansion and inflammation as well as the development of T2D-like features in FA animals. To validate these results, further analysis of the microbiota and adipokines in the feces and plasma of FA patients is warranted. These analyses on patients and the completion of the identification of the molecular mechanisms underlying the butyrate-mediated beneficial effects will hopefully pave the way for its safe usage as an adjuvant for treating T2D-related symptoms in FA.

## Limitation of the study

While this study contributes valuable insights into the metabolic implications of FA, certain limitations should be acknowledged. The findings were obtained from the KIKO model, which, while providing significant insights, may not fully represent the diverse spectrum of FA manifestations in humans. The observed effects in adipose tissue and metabolic pathways might be influenced by complex interactions between various tissues and systems that require further investigation. Additionally, the study primarily focused on the effects of dietary butyrate supplementation and its impact on vWAT dysfunction. Further research is needed to comprehensively understand the mechanisms underlying these effects and their potential translation to human FA patients. Moreover, while butyrate supplementation shows promise, its exact therapeutic dosage, safety profile, and long-term effects require careful assessment. These limitations warrant future studies involving larger animal models and clinical investigations to validate the observed benefits and to determine the broader applicability of butyrate-based interventions in managing FA-related metabolic complications.

## STAR★Methods

### Key resources table

**Table:** 

REAGENT or RESOURCE	SOURCE	IDENTIFIER
**Antibodies**		

VEGFA	Santa Cruz Biotechnology	Cat# sc-7269; RRID: AB_628430
HO-1	Abcam	Cat# ab13240; RRID: AB_2244379
IRP-1	Santa Cruz Biotechnology	Cat# sc-14216; RRID: AB_2223767
PPAR-γ	Santa Cruz Biotechnology	Cat# sc-7196; RRID: AB_654710
ATGL	Cell Signaling	Cat# 2439S; RRID: AB_2167953
Vinculin Monoclonal Antibody (VLN01)	ThermoFisher Scientific	Cat# MA5-11690; RRID: AB_10976821
FXN	Santa Cruz Biotechnology	Cat# sc-25820; RRID: AB_2110677
ACTIN	Proteintech	Cat# 20536-I-AP; RRID: AB_10700003
CD68	Abcam	Cat# ab125212; RRID: AB_10975465
PLIN1	Santa Cruz Biotechnology	Cat# sc-67164; RRID: AB_2252681
SOD1	Abcam	Cat# ab51254; RRID: AB_882757
PKA p-substrates	Cell Signaling	Cat# 9621; RRID: AB_330304
S100A8	Invitrogen	Cat# PA5-86063; RRID: AB_2802864
Goat anti-Mouse IgG (H + L) Cross-Adsorbed Secondary Antibody, Alexa Fluor 555, Thermo Fisher Scientific	ThermoFisher Scientific	Cat# A-21422; RRID: AB_141822
anti-CD45 magnetic beads-conjugated antibody	Miltenyi Biotec	Cat# 130-052-301; RRID: AB_2877061
CD11b APC-Vio770	Miltenyi Biotec	Cat# 130-113-232; RRID: AB_2726043
CD19 BV421	Biolegend	Cat# 115537; RRID: AB_10895761
CD3 PE	BD Biosciences	Cat# 552127; RRID: AB_394342
CD4 PerCP5.5	Biolegend	Cat# 100540; RRID: AB_893326
F4/80 FITC	Biolegend	Cat# 123107; RRID: AB_893500
Ly6C PE-Cy7	Biolegend	Cat# 128018; RRID: AB_1732082
Ly6G PE-CF594	BD Biosciences	Cat# 562700; RRID: AB_2737730
NK1.1 APC	Miltenyi Biotec	Cat# 130-120-507; RRID: AB_2752123

**Chemicals, peptides, and recombinant proteins**		

Sodium butyrate	Santa Cruz Biotechnlogy	Cat# 156-54-7
Oil Red O	Sigma-Aldrich	Cat# O0625-25G
3,3′,5-Triiodo-L-thyronine sodium salt	Sigma Aldrich	Cat# T6397
Rosiglitazone,≥98% (HPLC)	Sigma Aldrich	Cat# R2408
Insulin solution human	Sigma Aldrich	Cat# I9278
Collagenase, Type I, powder	ThermoFisher Scientific	Cat# 17100017
Collagenase, Type II, powder	ThermoFisher Scientific	Cat# 17101015
eBioscience™ 1X RBC Lysis Buffer	ThermoFisher Scientific	Cat# 00-4333-57
3-Isobutyl-1-methylxanthine,BioUltra, ≥99%	Sigma Aldrich	Cat# I7018
Lipopolysaccharides from Escherichia coli O111:B4,gamma-irradiated, BioXtra	Sigma Aldrich	Cat# L4391
Hoechst 33342	ThermoFisher Scientific	Cat# H3570
Applied Biosystems™ *Power*™ SYBR™ Green Master Mix	ThermoFisher Scientific	Cat# A25742
M-MLV Reverse Transcriptase	Promega	Cat# M1701
Recombinant Mouse MCSF (Animal-Free)	Cell Guidance Systems	Cat# GMF8AF
NAD^+^	Sigma Aldrich	Cat# 53-84-9
L-Lactic Dehydrogenase from bovine heart	Sigma Aldrich	Cat# L3916

**Critical commercial assays**		

Lipofectamine™ 2000 Transfection Reagent	ThermoFisher Scientific	Cat# 11668019
Seahorse XFe96 FluxPak	Agilent Technologies	Cat# 102416-100
Seahorse XF DMEM assay medium pack, pH 7.4	Agilent Technologies	Cat# 103680-100
OctoMACS™ Starting Kit	Miltenyi Biotec	Cat# 130-042-108
QuadroMACS™ Starting Kit (LS)	Miltenyi Biotec	Cat# 130-091-051
LS Columns	Miltenyi Biotec	Cat# 130-042-401
Pre-Separation Filters (30 μm)	Miltenyi Biotec	Cat# 130-041-407
RNeasy Lipid Tissue Mini Kit	Qiagen	Cat# 74804
Alexa Fluor™ 488 Phalloidin	ThermoFisher Scientific	Cat# A12379
DNA extraction kit	OMEGA, Bio-tek	Cat# M6399-01
Griess Reagent System	Promega	Cat# G2930
MACH 4 Universal HRP-Polymer Kit with DAB	Biocare Medical	Cat# G2930M4BD534 G, H, L
ECL Prime Western Blotting Reagent	GE Healthcare	Cat# RPN2232

**Experimental models: Cell lines**		

3T3-L1 cell line	ATCC	Cat# CL-173
RAW 264.7 cell line	ATCC	Cat# TIB-71

**Experimental models: Organisms/strains**		

C57BL/6J	The Jackson Laboratory	Cat# 000664
Knock-in knock-out (KIKO) mice	The Jackson Laboratory	Cat# 012329

**Oligonucleotides**		

FXN shRNA scramble shRNA	OriGene Technologies	Cat# TL514670V

**Software and algorithms**		

Fiji ImageJ	https://imagej.net/software/fiji/downloads	N/A
FastQC version 0.11.5	https://www.bioinformatics.babraham.ac.uk/projects/fastqc	N/A
Trimmomatic version 0.36	Bolger et al.8^86^	N/A
FluorChem FC3 Analysis Software	Bolger et al.8^86^	N/A
HISAT2 version 2.1.0	Kim et al.8^87^	N/A
StringTie version 1.3.4days	Pertea et al.8^88^	N/A
AnnotationDbi R library	http://bioconductor.org	N/A
Rosalind version 3.36.1.2	https://www.rosalind.bio/en/knowledge/how-does-rosalind-calculate	N/A
GAIA 2.0	https://metagenomics.sequentiabiotech.com/gaia/	N/A
GraphPad Prism 9	GraphPad Software Inc.	N/A

**Deposited data**		

16S rRNA gene sequencing data	This paper	ArrayExpress: E-MTAB-12871
RNAseq data	This paper	Gene Expression Omnibus: GSE239980

**Other**		

Glucometer	Bayer Countur XT	N/A
KeyLab analyser	BPCBioSed	N/A
NanoZoomer Digital Microscope	Hamamatsu Photonics	N/A
Olympus IX-81 confocal microscope	Olympus Life Science	N/A
Cytoflex	Beckman Coulter	N/A
API-3500 triple quadrupole mass spectrometer	AB Sciex	N/A
ExionLC™ AC System	AB Sciex	N/A

### Resource availability

#### Lead contact

Further information and requests for resources and reagents should be directed to and will be fulfilled by the lead contact: Katia Aquilano; e-mail: katia.aquilano@uniroma2.it.

#### Materials availability

This study did not generate unique reagents.

### Experimental model and study participant details

#### Animal experiments

Mouse experimentation was conducted in accordance with accepted standard of humane animal care after the approval by relevant local (Institutional Animal Care and Use Committee, Tor Vergata University) and national (Ministry of Health, licenses n° 324/218-PR and n° 210/202-PR) committees. Female and male mice were maintained at 21.0 ± °C and 55.0 ± 5.0% relative humidity under a 12 h/12 h light/dark cycle (lights on at 6:00 a.m., lights off at 6:00 p.m.). Food and water were given *ad libitum*. Experiments were carried out according to institutional safety procedures.

Female and male Knock-in knock-out (KIKO) mice were purchased from Jackson Laboratories (#012329). Female and male littermate C57BL/6 mice (WT) were used as controls. Researchers were blinded to genotypes at the time of testing. Butyrate supplementation was conducted in male mice according to Gao et al., 2009^25^ by adding sodium butyrate in food pellets (5 g · kg−1 · day−1 at the normal daily rate of calorie intake, beginning at 4 months of age, a stage where mice do not yet exhibit metabolic alterations, and continuing until 8 months of age (16 weeks treatment). This duration was chosen as it corresponds to the point at which mice start displaying metabolic alterations and weight gain.^8^^,^^9^

Mice were sacrificed by cervical dislocation at 8 months of age. vWAT tissues were explanted, immediately processed or stored at −80°C.

#### Cell culture

Murine 3T3-L1 cell line were purchased from ATCC (Manassas, VA, USA), and cultured and differentiated in adipocytes according to ATCC protocol. Oil Red O was used to detect intracellular triglycerides. Briefly, adipocyte cultures were fixed with 4% formaldehyde for 30 min. After rinsing with distilled water, cells were stained for 30 min at room temperature with Oil Red O working solution, prepared by diluting a stock solution (0.5% Oil Red O in isopropanol) with distilled water in a 3:2 ratio. After removal of excess stain with distilled water, neutral lipids were quantified after extraction with 4% IGEPAL in isopropanol followed by 550 nm absorbance analysis.

Murine RAW 264.7 macrophages (ATCC) were cultured in DMEM supplemented with 10% FBS and 1% P/S (Life Technologies). All cells were maintained at 37°C in a humidified incubator containing 5% CO_2_.

For gene silencing, mature 3T3-L1 adipocytes were transfected with FXN shRNA scramble shRNA (Origene, Rockville, MD, USA), using LipofectamineTM 2000 transfection reagent (ThermoFisher) according to manufacturer’s instructions. Cells were used 48 h after transfection.

Twenty-four hours after plating, RAW264.7 cells were used for the experiments such as co-culturing with white adipocytes or treatment with 500 ng/mL LPS for 16 h.

Sodium butyrate was used at concentration of 500 μM for 16 h. When used in co-treatment with LPS, butyrate was added in the culture medium 1 h prior to LPS and maintained throughout the experiment. The sodium butyrate concentration was selected based on dose-response experiments conducted on primary adipocytes or BMDM stimulated with LPS (500 ng/mL, 16 h). These experiments demonstrated the anti-inflammatory action of the 500 μM concentration while preserving cell viability.

### Method details

#### Bio-clinical analyses

Prior to bio-clinical analyses, male mice were starved for 12 h. After blood collection, bio-clinical analyses were performed by colorimetric methods. In particular, cholesterol, triglyceride, GOT, GPT, LDH and creatinine levels were measured in plasma through the automatized KeyLab analyser (BPCBioSed, Italy) using specific assay kits (BPCBioSed).

For the glucose tolerance test (OGTT), male mice were subjected to fasting for 12 h, followed by oral gavage with 2 g of dextrose/kg body mass. At the indicated time points, blood was collected from the tail vein and glycemia measured using a glucometer (Bayer Countur XT, Bayer Leverkusen, Germany).

#### Histochemical and immunohistochemistry analysis

vWAT was stained with H&E or Trichrome staining to visualize general morphology and collagen deposition, respectively. For morphometric analyses, individual slides were digitized using the NanoZoomer Digital Microscope (Hamamatsu, Japan), and digital images were analyzed using ImageJ to measure the diameter of adipocytes. Values are the means of 10 fields taken from different tissue sections per mouse. Immunohistochemical detection of VEGFA or S100A8 was performed on 3- to 5-lm-thick sections obtained from formalin-fixed tissue embedded in paraffin. Antigen retrieval was performed with Citrate Buffer (pH 6) (Dako, Glostrup, Denmark). Immunohistochemical staining was performed with anti VEGFA or anti-S100A8. Incubations with primary antibodies were carried out for 2 h. Negative controls were obtained by omitting primary antibodies. The immunohistochemical procedure was performed using the MACH 4 Universal HRP-Polymer Kit with DAB as chromogen (Biocare Medical, Concord, CA, USA).

For quantification of fibrosis area with Trichrome staining, image analysis using Qpath and ImageJ (open source) was performed in four randomly selected 10X fields. For immunohistochemistry, quantification of the percentage of positive cells in four randomly selected 5X fields was performed with Qpath.

#### Immunofluorescence analyses

Sections of frozen vWAT were incubated with permeabilization solution (PBS/Triton X-100 0.2% [v/v]), blocked for 1 h by a blocking solution (PBS/BSA 5% [v/v]), and then incubated for 18 h with CD68 or VEGFA primary antibodies. After washing with cold PBS, sections were incubated 1 h with Alexa Fluor 488 or -568-conjugated secondary antibodies (ThermoFisher Scientific). Nuclei were stained with 10 μg/mL Hoechst 33342 (ThermoFisher Scientific). Alexa Fluor 488 Phalloidin (ThermoFisher Scientific) was used to stain actin. Images were acquired using an Olympus IX-81 confocal microscope at 60× magnitude. Representative regions of interest were acquired using a digital 3× zoom. Fluorescence intensities were set for the control samples and were maintained for all samples. To evaluate macrophage infiltration, CD68^+^ cells around adipocytes were counted in each field (10 fields/sample).

#### Immunoblotting

Tissues or cells were lysed in RIPA buffer (50 mM Tris-HCl, pH 8.0, 150 mM NaCl, 12 mM deoxycholic acid, 0.5% Nonidet P-40, and protease and phosphatase inhibitors). Five μg proteins were loaded on SDS-PAGE and subjected to Western blotting. Nitrocellulose membranes were incubated with primary antibodies at 1:1000 dilution. Successively, membranes were incubated with the appropriate horseradish peroxidase-conjugated secondary antibodies. Immunoreactive bands were detected by a FluorChem FC3 System (Protein-Simple, San Jose, CA, USA) after incubation of the membranes with ECL Prime Western Blotting Reagent (GE Healthcare, Pittsburgh, PA, USA). Densitometric analyses of the immunoreactive bands were performed by the FluorChem FC3 Analysis Software.

#### Bulk RNA-sequencing and functional enrichment analysis

Total vWAT RNA was isolated using TRIzol reagent (Invitrogen, Waltham, MA, USA) and purified using the RNeasy mini kit protocol (Qiagen, Hilden, Germany) according to the manufacturer’s instructions. Isolated RNA was sequenced using an Illumina NextSeq500, and the indexed libraries were prepared from 1 μg of purified RNA with TruSeq-stranded mRNA (Illumina) Library Prep Kit according to the manufacturer’s instructions. The quality of the single-end reads was evaluated using FastQC version 0.11.5 (https://www.bioinformatics.babraham.ac.uk/projects/fastqc). All FastQC files were filtered to remove low-quality reads and adapters using Trimmomatic version 0.36.^86^ The resulting reads were mapped to the *Mus musculus* genome (GRCm38) using HISAT2 version 2.1.0^87^ using default parameters, and StringTie version 1.3.4days^88^ was applied to the BAM files obtained using HISAT2 to generate expression estimates and to quantify the transcript abundance as transcripts per kilobase per million of mapped reads. The count matrices generated by StringTie were imported in R, in which differential expression analysis was performed using Deseq2 to compare the two different conditions. Functional annotation was performed using the AnnotationDbi R library (http://bioconductor.org). Differentially expressed genes were selected with a threshold of FC > 1.5 or <0.5 (FDR <0.05). Functional enrichment analyses were performed using Rosalind version 3.36.1.2.

#### RT-qPCR

Total RNA was extracted using TRI Reagent (Sigma-Aldrich). RNA (3 μg) was retro-transcribed by using M-MLV (Promega, Madison, WI). qPCR was performed in triplicate by using validated qPCR primers (BLAST), Applied Biosystems *Power* SYBR Green Master Mix, and the QuantStudio3 Real-Time PCR System (ThermoFisher, Whaltam, MA, USA). mRNA levels were normalized to actin mRNA, and the relative mRNA levels were determined through the 2^−ΔΔCt^ method. The primers used for RT-qPCR are listed below:

*Adipoq* FWD 5′-GACCTGGCCACTTTCTCCTC-3′ REV 5′-TCCTGAGCCCTTTTGGTGTC-3’

*Cox2* FWD 5′-CCTCCATTGACCAGAGCAGAG-3′ REV 5′-AGCCATTTCCTTCTCTCCTGTAA-3’

*Fxn* FWD 5′-TCTCTTTTGGGGATGGCGTG-3′ REV 5′-GCTTGTTTGGGGTCTGCTTG-3’

*Il10* FWD 5′-GCAGGACTTTAAGGGTTACTTGG-3′ REV 5′-GGGGCATCACTTCTACCAGG.

*Il1b* FWD 5′-TGCCACCTTTTGACAGTGATG-3′ REV 5′-AAGGTCCACGGGAAAGACAC-3’

*Il6* FWD 5′-GGATACCACTCCCAACAGA-3′ REV 5′-GCCATTGCACAACTCTTTTCTCA-3’

*Pparg* FWD 5′-CGCGGAAGAAGAGACCTGG-3′ REV 5′-ACCGCTTCTTTCAAATCTTGTCTG.

*Rasip* FWD 5′-CTCACTGATGGAACGAGGTCAA-3′ REV 5′-CCCCTGAAGCCAATCCAACA-3’

*Roboa4* FWD 5′-AGGCCAAAAAGAAGCAGGAATTG-3′ REV 5′-CTTCTCCTGGGGAACCAGAGT-3’

*Vegfa* FWD 5′-AGCAGATGTGAATGCAGACCA-3′ REV 5′-ACCGCTTCTTTCAAATCTTGTCTG-3’

#### Isolation of stromal vascular cells and bone marrow derived macrophages

Stromal vascular cells (SVCs) were obtained by finely mincing vWAT of 8 months-old female and male mice with scissors, followed by incubation in high-glucose DMEM containing 0.1% collagenase II at 37°C in an orbital shaker (150 rpm, 1h). The resulting cell suspension was then filtered through a 100-μm nylon mesh to remove any remaining tissue fragments. The filtered cells were collected in a 50-mL conical tube and centrifuged at 500 × g for 5 min at 4°C to remove any supernatants and floating adipocytes.

Pellet was resuspended in 1 mL of ACK RBC lysis buffer and incubated at room temperature for 2 min to remove any remaining red blood cells. The RBC lysis reaction was then quenched by adding 10 mL of cold wash media (high-glucose DMEM containing 5% heat-inactivated FBS, L-glutamine, and penicillin/streptomycin). The cells were then centrifuged again at 500 × g for 5 min at 4°C, and the resulting pellets containing the SVCs were collected.

For isolation of bone marrow derived macrophages (BMDMs), bone marrow was extracted from the limbs of 8-months-old female mice by perfusion with PBS and 1% P/S. BMDMs were plated at a density of 3 × 10^5^ cells/mL in alpha-MEM supplemented with 10% FBS, 1% P/S, and 1% GlutaMAX. Macrophage differentiation was induced by adding M-CSF (20 ng of cells/mL) in the culture medium for 5 days. Following adhesion, unattached cells were removed, and BMDM were used for the experiments.

#### Magnetic cell sorting

SVCs were resuspended in 500 mL of magnetic bead buffer (MBB) consisting of PBS without calcium and magnesium, 0.5% w/v bovine serum albumin (BSA), and 2 mM ethylenediaminetetraacetic acid (EDTA). The cell suspension was then filtered through a 30-mm pre-separation filter (Miltenyi, Bergisch Gladbach, Germany) following three filter washes to remove any large particles and debris.

The resulting cell suspension was then separated at 300 × g for 5 min at 4°C and resuspended in 90 mL of MBB along with 10 mL of anti-CD45 magnetic beads-conjugated antibody (Miltenyi) for each 10ˆ7 cell sample. The cell suspension was incubated for 15 min at 4°C, then diluted with 2 mL of MBB and centrifuged. The resulting cell pellet was resuspended in 500 mL of MBB, applied onto hydrated MS-columns (Miltenyi), washed three times with 500 mL of MBB, and collected with 1 mL of MBB through piston elution.

#### High dimensional flow cytometry

SVCs were stained on a polystyrene 96-well V bottom tissue-culture treated plate. The cells were centrifuged at 500 × *g* for 5 min at 4°C, and the cell pellet was washed in PBS once and centrifuged again as described above. The cells were incubated with 25–50 μL of FcBlock (1:100, BD Pharmigen in FACS buffer) on ice for 10 min, and then an equal volume of the staining cocktail was added and mixed. For the identification of the main infiltrated leukocyte populations, total leukocytes were identified gating on CD45^+^ cells. Inside this gate, neutrophils were identified as Ly6G^+^Ly6C^−^ cells and macrophages as CD11b+F4/80+CD64^+^ cells. Ly6C^−^Ly6G^−^ cells were further gated to identify CD3^+^ T-lymphocytes, NK1.1^+^ NK cells and CD19^+^ B-lymphocytes. Samples were acquired on a 13-color Cytoflex (Beckman Coulter) and for each analysis, at least 0.5 × 10^6^ live cells were acquired by gating on aqua Live/Dead negative cells and upon exclusion of cell doublets.^89^^,^^90^^,^^91^

#### Lactate and nitrite assay

Extracellular lactate was measured in culture medium by an enzyme-based spectrophotometric assay. Cell media were collected and treated with 1:2 (v/v) 30% trichloroacetic acid to precipitate proteins. The resulting mixture was then centrifuged at 14,000×g for 20 min at 4°C, and the supernatant was collected and incubated (30 min, 37°C) with a reaction buffer containing glycine, hydrazine, NAD^+^ and LDH enzyme to allow the conversion of lactate to pyruvate, while simultaneously reducing NAD^+^ to NADH. NADH concentration, stoichiometrically equivalent to the amount of lactate, was determined at 340 nm using a spectrophotometer (extinction coefficient of 6220 M^−1^ cm^−1^). Lactate concentrations were normalized to the total protein content of cell lysates measured by Lowry method.

Nitrite concentration was determined by Griess reaction using a Griess Reagent System (Promega).

#### Seahorse analysis

Cells were seeded at 1.5 × 10^4^ cells/well in a Seahorse XF 96-well plate. Mito-stress test and glycolysis test were performed according to Agilent Technologies recommendations. Oxygen consumption rate (OCR) and extracellular acidification rate (ECAR) was used as an indicator of mitochondrial function and glycolytic activity, respectively. Analyses were performed using the Desktop Wave Software (Agilent Technologies).

#### Targeted metabolomics

Metabolomic data were obtained using liquid chromatography coupled to tandem mass spectrometry. We used an API-3500 triple quadrupole mass spectrometer (AB Sciex, Framingham, MA, USA) coupled with an ExionLC AC System (AB Sciex). vWAT specimens were disrupted in a tissue lyser for 1 min at maximum speed in 250 μL ice-cold methanol:acetonitrile 1:1 (v/v) containing 1 ng/μL [U-^13^C_6_]-glucose and 1 ng/μL [U-^13^C_5_]-glutamine as internal standards. Lysates were spun at 15,000 g for 15 min at 4°C. Samples were then dried under N_2_ flow at 40°C and resuspended in 5 mM ammonium acetate in methanol:water 1:1 (v/v) for subsequent analyses.

Quantification of amino acids, their derivatives, and biogenic amines was performed through previous derivatization.^92^ Briefly, 25 μL of each 125 μL sample were collected and dried separately under N_2_ flow at 40°C. Dried samples were resuspended in 50 μL phenyl-isothiocyanate, EtOH, pyridine, and water 5%:31.5%:31.5%:31.5%, then incubated for 20 min at RT, dried under N_2_ flow at 40°C for 90 min, and finally resuspended in 100 μL 5 mM ammonium acetate in MeOH/H_2_O 50:50. Quantification of different amino acids was performed using a C18 column (Biocrates, Innsbruck, Austria) maintained at 50°C. The mobile phases for positive ion mode analysis were phase A: 0.2% formic acid in water and phase B: 0.2% formic acid in acetonitrile. The gradient was T_0_: 100%A, T_5.5_: 5%A, T_7_: 100%A with a flow rate of 500 μL/min. All metabolites analyzed in the described protocols were previously validated by pure standards and internal standards were used to check instrument sensitivity.

Quantification of energy metabolites and cofactors was performed using a cyano-phase LUNA column (50 mm × 4.6 mm, 5 μm; Phenomenex) with a 5.5 min run in negative ion mode with two separated runs. Protocol A: mobile phase A was water and phase B was 2 mM ammonium acetate in MeOH, with a gradient of 10% A and 90% B for all analyses and a flow rate of 500 μL/min. Protocol B: mobile phase A was water and phase B was 2 mM ammonium acetate in MeOH, with a gradient of 50% A and 50% B for all analyses and a flow rate of 500 μL/min.

Acylcarnitine quantification was performed on the same samples using a Varian Pursuit XRs Ultra 2.8 Diphenyl column (Agilent). Samples were analyzed in a 9 min run in positive ion mode. Mobile phases were A: 0.1% formic acid in H_2_O, B: 0.1% formic acid in MeOH, and the gradient was T_0_: 35%A, T_2.0_: 35%A, T_5.0_: 5%A, T_5.5_: 5%A, T_5.51_: 35%A, T_9.0_: 35%A with a flow rate of 300 μL/min.

MultiQuant software (version 3.0.3, AB Sciex) was used for data analysis and peak review of chromatograms. Raw areas were normalized to the areas’ median. Obtained data were then compared to controls and expressed as fold change. Raw data are reported in Table S1.

#### 16s sequencing analysis

Upon collection, fecal pellets were immediately preserved by freezing in liquid nitrogen and then stored at −80°C. To extract fecal nucleic acid, an E.Z.N.A. stool DNA kit (OMEGA, Bio-tek) was used. The bacterial 16S rRNA gene was amplified from total DNA following the Illumina 16S Metagenomic Sequencing Library Preparation instructions, targeting the V3-V4 hypervariable region amplicon by PCR with universal primers containing Illumina adapters reported in Klindworth et al.^93^ The resulting amplicon was purified and subjected to a second PCR to barcode the libraries using the Illumina dual-index system before a final purification step. The pooled libraries were then sequenced using paired-end sequencing (2 × 300 cycles) on an Illumina MiSeq device according to the manufacturer’s specifications. The resulting sequence data obtained as FASTq files were analyzed using 16S Metagenomics GAIA 2.0 software, which performs quality control on the reads/pairs (i.e., trimming, clipping, and adapter removal) through FastQC and BBDuk before mapping them with BWA-MEM against NCBI databases. The average number of reads per sample was 220,507.1 (SD ± 104,675.1).

### Quantification and statistical analysis

#### Statistical analysis

Data were expressed as the mean ± SD. The exact numbers of replicates are given in each figure legend. A two-tailed unpaired Student’s *t* test was performed to assess the statistical significance between two groups. ANOVA analysis of variance followed by Dunnett’s (comparisons relative to controls), or Tukey’s (multiple comparisons among groups) *post hoc* tests was used to compare three or more groups. Statistical analyses were performed using GraphPad Prism 9 (GraphPad Software Inc., San Diego, CA, USA). In all cases, a p value of 0.05 was set as the significance threshold.

## Data Availability

• Raw 16S rRNA gene sequencing data are publicly accessible through ArrayExpress; RNAseq data have been deposited on Gene Expression Omnibus and are publicly accessible as of the date of publication. Accession numbers of deposited datasets are listed in the key resources table. • This paper does not report original code. • Any additional information required to reanalyze the data reported in this paper is available from the lead contact upon request.

## Supplemental information

Document S1. Figure S1

Table S1. Targeted metabolomic analysis results, related to Figure 6. Raw areas for each metabolite are reported
