# Supplementary material for: Butyrate prevents visceral adipose tissue inflammation and metabolic alterations in a Friedreich’s ataxia mouse model
Source: iScience. 2023 Aug 28;26(10):107713. doi: 10.1016/j.isci.2023.107713 (PMC10494209; doi:10.1016/j.isci.2023.107713)
Supplement: Document S1. Figure S1 [file mmc1.pdf]

iScience, Volume *26*

Supplemental information

Butyrate prevents visceral adipose tissue inflammation and metabolic alterations in a Friedreich's ataxia mouse model

Riccardo Turchi, Francesca Sciarretta, Veronica Ceci, Marta Tiberi, Matteo Audano, Silvia Pedretti, Concetta Panebianco, Valentina Nesci, Valerio Pazienza, Alberto Ferri, Simone Carotti, Valerio Chiurchiù, Nico Mitro, Daniele Lettieri-Barbato, and Katia Aquilano

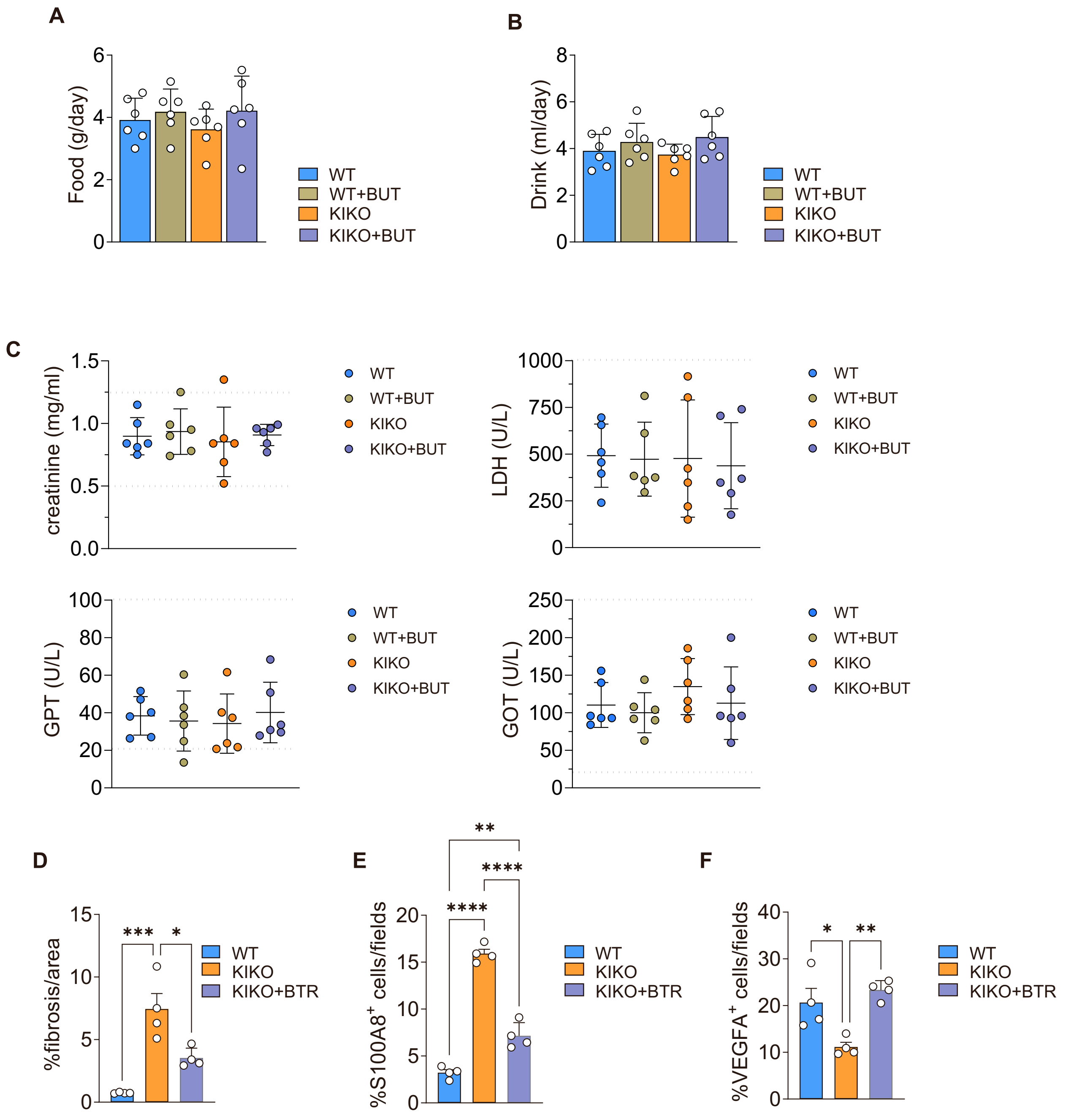

Figure S1. Butyrate supplementation does not affect mouse behaviour and tissue function and prevents vWAT alteration in KIKO mice, related to Figure 4 and Figure 5. Four-months male WT and KIKO mice were fed with normal diet or with diet supplemented with butyrate (+BTR) for 16 weeks (up to 8-months age). **A, B**) Feeding and drinking behaviour following butyrate supplementation. Data are expressed as mean ± SD (n=6 male mice/group); **C**) Analysis of plasma levels of creatinine, LDH, GOT and GPT. Dashed lines indicate the normal values of the analysed bio-clinical parameters. Data are expressed as mean ± SD (n=6 male mice/group); **D-F**) Quantification of fibrosis (D), immune cell infiltration (E) and VEGFA expression (F) in vWAT. Data are expressed as mean ± SD (n=4 male mice/group; ANOVA test, *p<0.05, **p<0.01, ***p<0.001, ****p<0.0001).
